# An Evaluation of Treatment Outcomes in a Cohort of Clients on the DOTS Strategy, 2012–2016

**DOI:** 10.1155/2018/4287842

**Published:** 2018-02-15

**Authors:** Ato Kwamena Tetteh, Edward Agyarko, Joseph Otchere, Langbong Bimi, Irene Ayi

**Affiliations:** ^1^Laboratory Department, Metropolitan Hospital, P.O. Box 174, Cape Coast, Ghana; ^2^Department of Community Medicine and Health, Anglican University College of Technology, P.O. Box 74, Nkoranza, Ghana; ^3^Noguchi Memorial Institute for Medical Research, Department of Parasitology, College of Health Sciences, University of Ghana, Legon, Accra, Ghana; ^4^Department of Animal Biology and Conservation Sciences, University of Ghana, Legon, Accra, Ghana

## Abstract

We present, for the first time, an evaluation of treatment outcomes in a cohort at a TB referral centre in the Central Region of Ghana. Of the 213 clients placed on DOTS, 59.2% (126/213) were sputum smear-positive. An overall cure rate of 90.2% (51.6% cured + 37.6% completed) and a death rate of 8.5% (18/213) were estimated. Of the number of clients who died, 5.7% (12/213) were males (*χ* ^2^ = 2.891, *p* = 0.699; LR = 3.004, *p* = 0.699). Deaths were only recorded among clients who were > 19 years old (*χ* ^2^ = 40.319, *p* = 0.099; LR = 41.244, *p* = 0.083). Also, 0.9% (2/213) was lost to follow-up, while 1.4% (3/213) had treatment failure. In total, 13.6% (7.0%, 15/213 males, and 6.6%, 14/213 females) of clients who were placed on DOTS were HIV seropositive. Ages of 40–49 years had the highest number, 13/213 (6.1%), infected with HIV, though the difference among the remaining age groups was not statistically significant (*χ* ^2^ = 9.621, *p* = 0.142). Furthermore, 7.0% (15/213) had TB/HIV coinfection. Out of them, 9 were cured and 5 died at home, while 1 had treatment failure. Tuberculosis/HIV infection prevention advocacy and interventions that address sociodemographic determinants of unfavourable treatment outcomes are urgently required to augment national efforts towards control.

## 1. Introduction

Successful tuberculosis (TB) treatment has a positive significant effect on the control of TB. Therefore, completing prescribed medication in active cases is of prime importance to TB control programmes [1]. Globally, the Directly Observed Therapy Short-course (DOTS) has been accepted as a strategy to the cure of tuberculosis [2]. The DOTS strategy has been used in Ghana since 1994, and it remains the medication strategy of choice [3]. This strategy aims at improving patients' commitment to medication and therefore minimizes development of drug resistance to contemporary remedies [4]. With this strategy, it is expected that 80 to 90% of patients will undergo smear conversion within two to three months of treatment [5].

The change in bacterial infection status of sputum of patients from initial Acid-Fast Bacilli (AFB) positive to negative after treatment is referred to as sputum smear conversion (SSC). Smear conversion rate (SCR) at two months of intensive phase (IP) and at three months of IP (extended IP) is a significant operational indicator, as it shows the capacity of the programme to maintain the patients on treatment. It also provides an objective verification for the client's response to therapy and consequently the treatment outcome [5].

The Global TB Report (2011) estimated 86 smear-positive pulmonary TB cases per 100,000 people per year [6]. It has been established that one untreated infectious tuberculosis patient is likely to infect 10 to 15 persons annually [7]. Also, a number of factors have been acknowledged as delaying the time to smear and culture conversion. These include high intensity initial sputum smear AFB grade, cavitary lesion, uncontrolled hyperglycaemia/diabetes mellitus, old age, certain ethnic population (e.g., Hispanic), multidrug-resistant tuberculosis, initial treatment with less than four antitubercular drugs, and non-rifampicin-based treatment regimens [8–14].

Generally, only sputum culture confirms noncontagious clients. However, lack of culture facilities in most TB centres, except for Regional/Teaching Hospitals in Ghana, necessitates the use of serial negative smears (SNS) before removal of infection control measures. Sputum examination has been described worldwide as the most definite, cost-effective, and dependable test for the diagnosis of pulmonary TB. It confirms the diagnosis and provides information on whether there is positive and progressive response to treatment. At the end of treatment, it shows if clients have been cured. The Metropolitan Hospital (MH), located within the Cape Coast Metropolitan Area (CCMA), has a TB testing centre and it is the only TB referral centre in the Central Region of Ghana. Yearly, about 250–400 new diagnostic cases are referred for testing from within CCMA and adjoining communities. A third of these return test results to treatment centres within their communities for management. The remaining, who reported to MH for consultation, may be recruited onto the DOTS strategy, which is comprehensively elaborated in the Tuberculosis Case Management Desk Aide. Over the past decades, no on-site assessment has been done regarding the conversion rates and performance of the current DOTS strategy. This study presents the outcome of a five-year retrospective evaluation of TB-confirmed and TB-suggestive clients placed on the DOTS strategy.

## 2. Materials and Methods

### 2.1. Setting

**Figure 1:**
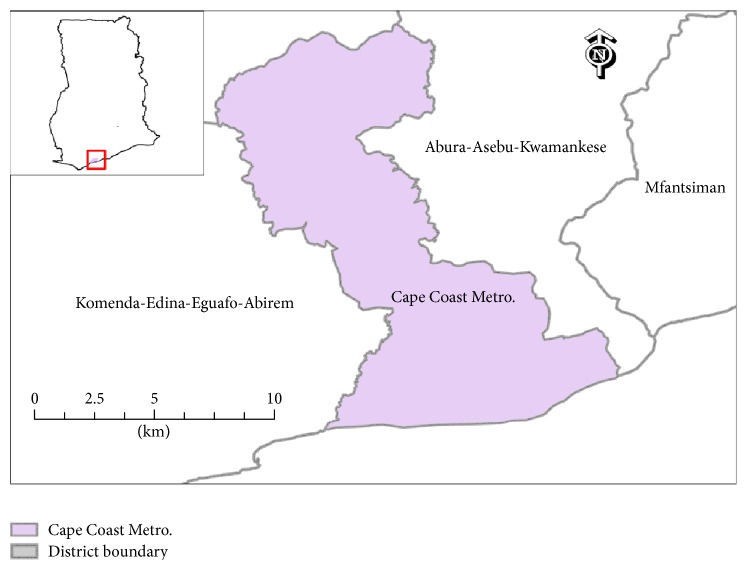
*Map of Cape Coast Metropolitan Area (credit: Dr. Charles Gyamfi, Department of Civil Engineering, Tshwane University of Technology, Pretoria, South Africa)*.

The entire Cape Coast Metropolis covers an area of 122 km^2^ and it is located on longitude 1°15′W and latitude 5°06′N (Figure 1). The Metropolitan Hospital, Cape Coast, was established in 1939 and it is located on the Beulah Road (Beach Road) between the Nursing and Midwifery Training College and St. Augustine's College. The hospital serves the entire Metropolis and it is the first referral facility for all Government Health Centres and Clinics in the area, as well as the adjoining communities (https://www.google.com.gh/maps/@5.1038147,-1.2596938,587m/data=!3m1!1e3). The hospital has a separate special structure, christened “Chest Ward,” where all clients diagnosed or suspected of having TB are admitted.

### 2.2. Study Design and Population

This was a five-year retrospective study comprising all diagnosed and suspected clients who were admitted and enrolled on the DOTS strategy from 1 January 2012 to 31 December 2016 (Table 1). In total, 213 male and female clients of all ages were included in the study. Compiled data included clients who were acid fast bacilli- (AFB-) positive or negative. All clients had persistent cough, with/without expectoration, increased axillary temperature, loss of appetite, and weight loss.

### 2.3. Data Collection

Secondary data were compiled from TB patients' registers at the Chest Ward. Client information collected included age, sex, form of tuberculosis (pulmonary or extrapulmonary), type of tuberculosis (smear-positive or smear-negative), category of tuberculosis (new cases or relapse or retreatment cases), and treatment outcome. Treatment outcome of clients was evaluated in harmony with World Health Organization reference and classified as cured, completed (treatment completed), default, treatment failure, death, and others. In this study, treatment was based on pretreatment sputum smear grading (PSSG) and/or TB-suggestive symptoms/sign, coupled with X-ray suggestive images. In addition, the HIV statuses of TB clients were confirmed from their medical records. Those whose status was not already known were tested using the preliminary First Response HIV® 1/2 and the confirmatory OraQuick® HIV 1/2.

### 2.4. Treatment Regimen

 See Table 1.

### 2.5. Ethical Approval

Permission was sought from the Metropolitan Hospital and the Chest Centre to access TB clients' archived data. Path numbers of patients were not recorded at all in order to conceal their identity. It was emphasized that data presented in this study will strictly be for academic purposes only to help improve management of patients in the future and will cause no harm in accordance with the Declaration of Helsinki (1964).

### 2.6. WHO Operational Definitions

*New smear-positive TB case* is a patient who has never had treatment for TB or who has taken anti-TB drugs for less than one month.* Treatment outcome* was divided into six categories according to the National TB Control Programme guidelines:* cured*: a patient who was smear-positive at diagnosis and was smear-negative at the end of the last month of treatment and on at least one previous occasion;* treatment completed*: any patient who was smear-negative but with suggestive signs/symptoms of TB at the time of diagnosis and had completed treatment, with or without sputum smear results;* died*: any patient who died irrespective of the cause of death during the course of treatment;* treatment failure*: any patient who remained or became positive at the end of the fifth month or later during treatment;* treatment defaulter*: any patient who has interrupted treatment two consecutive months or more after the date of the last attendance during the course of treatment;* transfer-out*; a patient who was transferred to another treatment centre and whose treatment outcome is unknown.

### 2.7. Data Analysis

All records from TB patients' registers from 2012 to 2016 were recoded, concealing the identities of clients. Data were double-entered into Microsoft Excel® and then cleaned (checked and edited) of inconsistencies. Completeness of the TB patients' registers after cleaning was approximately 99.5%. Data were later transported into SPSS (version 20) for analysis. Results were expressed as absolute frequencies (*n*) and percentages (%). Proportions with 95% confidence intervals and Chi-square test were employed to compare different groups and *p* value less than 0.05 was considered statistically significant. Smear conversion was estimated as the time (in weeks) from initiation of treatment to first of the two serial negative smears for AFB. Cure rate was deduced according to the WHO cure rate/treatment success rate definition as follows: the proportion of new smear-positive TB cases registered under DOTS in a given year that successfully completed treatment, whether with (“cured”) or without (“treatment completed”) bacteriologic evidence of success. The study consolidated the respective yearly data to obtain overall rates for the period under review.

## 3. Results

### 3.1. Sociodemographic Characteristics

In total, 213 clients whose data were available in tuberculosis (TB) treatment registers from 2012 to 2016 (2012 = 44; 2013 = 55; 2014 = 48; 2015 = 44; 2016 = 22) were included in the analysis. These consisted of 54 females and 159 males (*χ* ^2^ = 16.9, *p* = 0.013), aged between 4 and 79 years (mean age = 42.37 ± 15.33) (Table 2). Of the total, 38.0% were from Cape Coast, while 62.0% were from adjoining rural communities.

### 3.2. Pretreatment Sputum Smear Grade and Treatment Outcome among Clients

Of the total number of clients on DOTS, 40.8% (87/213) were sputum smear-negative (SSN), while the remaining 59.2% (126/213) were sputum smear-positive (SSP) (Table 3). In all, 173 were placed in category I, 38 in category II, and 2 in category III (table not shown). Percentage distribution of the pretreatment sputum smear grade (PSSG) among clients is as indicated in Table 4. Out of the total SSP clients, 77.0% (97/126) were males and 23% (29/126) were females. The overall cure rate, 90.2%, was determined by adding the percentage cured to the percentage that completed the treatment.

### 3.3. Death Rate for TB Clients from 2012 to 2016

According to the records, two clients were lost to follow-up (Table 3). Of these, one was SSN, while the other was SSP. In total, 8.5% (18/213) of clients on DOTS died (17 at home and 1 at the Chest Ward) during the period under review. The annual death distribution over the entire period is as follows: 2012, 0.5% (1/213); 2013, 1.9% (4/213); 2014, 2.8% (6/213); 2015, 2.4% (5/213); and 2016, 0.9% (2/213). Among the sexes, six females (2.8%) died compared to 12 males (5.7%) (*χ* ^2^ = 2.891, *p* = 0.699; likelihood ratio = 3.004, *p* = 0.699). With regard to the age groups, no death was recorded among clients ≤ 19 years; in the age group of 20–29 years, one death was recorded; in the age group of 30–39 years, three deaths were recorded; in the age group of 40–49 years, six deaths were recorded; in the age group of 50–59 years, six deaths were recorded; and in the age group of ≥60 years, one death was recorded (*χ* ^2^ = 40.319, *p* = 0.099; likelihood ratio = 41.244, *p* = 0.083).

### 3.4. HIV-Infected TB Clients on DOTS from 2012 to 2016

In total, 13.6% (29/213) who were HIV seropositive were placed on DOTS (Table 4). This was based on either a positive PSSG or a TB-suggestive sign/symptom, coupled with an X-ray image. Although more males were placed on DOTS as seen in the entire evaluation, an approximately equal number of males (7.0%, 15/213) and females (6.6%, 14/213) were found to be infected with HIV. Among the age groups, the age group of 40–49 years had the highest number (6.1%, 13/213) of HIV infections; however, the distribution among the remaining age groups was statistically insignificant (*χ* ^2^ = 9.621, *p* = 0.142, likelihood ratio = 9.584, *p* = 0.143). The annual trend of HIV infection among clients on DOTS for the period is as indicated in Table 4 (*χ* ^2^ = 2.742, *p* = 0.602, likelihood ratio = 2.995, *p* = 0.559).

### 3.5. Treatment Outcome of TB/HIV Coinfected Clients

**Figure 2:**
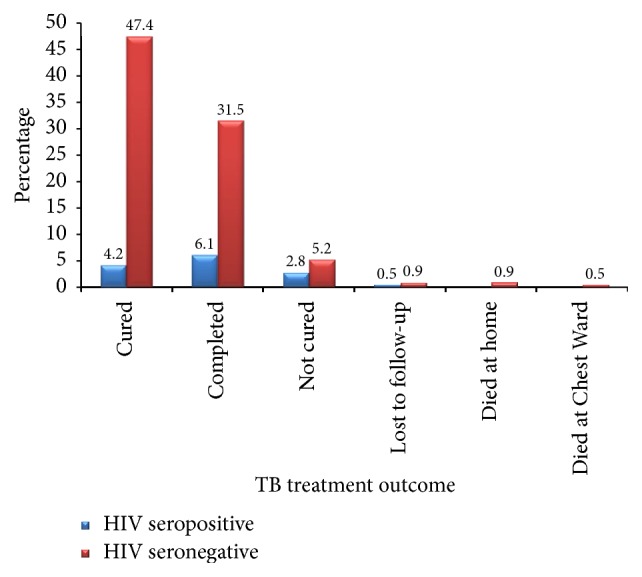
*Treatment outcomes for TB/HIV coinfected clients*.

Pretreatment sputum smear grading among these HIV-infected clients showed that 14 out of the 29 (48.3%) infected had no AFB. The remaining 15 were coinfected with TB. Of the 29 clients with TB and HIV coinfection, nine were cured, 13 completed the treatment, and 6 were not cured, while 1 was lost to follow-up (Figure 2). The fate of the one who was lost to follow-up could not be traced in the TB register. None of the clients with the HIV infection died during the period under review.

### 3.6. Analysis on TB/HIV Coinfection

**Figure 3:**
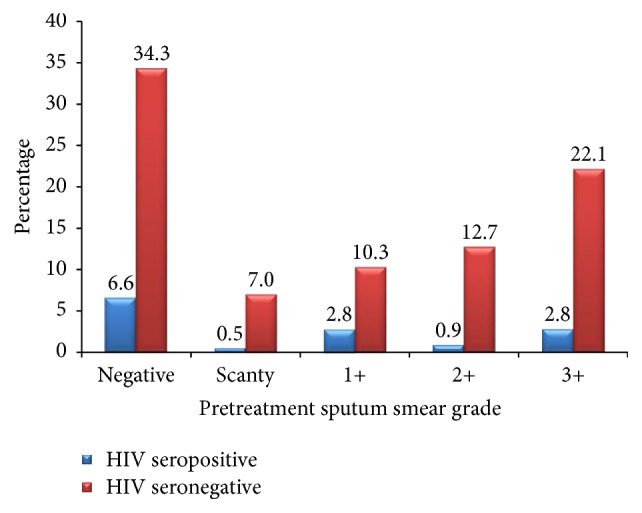
*TB/HIV coinfection*. Negative:* no Acid-Fast Bacilli (AFB) seen in at least 100 fields*; scanty (sc):* 1–9 AFB found in 100 fields*; (1+):* 10–99 AFB found in 100 fields*; (2+):* 1–10 AFB found per field in at least 50 fields*; (3+):* more than 10 AFB per field in at least 20 fields*.

Out of the 126 clients whose PSSG was positive (sc, 1+, 2+, or 3+), 15 were coinfected with HIV. This number represents 7.0% (15/213) of the total number of clients evaluated. The distribution of TB/HIV coinfection with regard to PSSG is as shown in Figure 3. With regard to their treatment outcome, 9 got cured and 5 died at home, while 1 had treatment failure (not cured). There was no default among the TB/HIV coinfected clients.

## 4. Discussion

A comprehensive evaluation of treatment outcomes is of supreme importance to the National TB Control Programme. Significant among strategic schemes to reduce the prevalence of TB infection worldwide is the annual evaluation of treatment outcomes in sputum smear-positive TB cases and among individuals with TB/HIV coinfection. This study sought to do that evaluation.

Out of the total, 51.6% clients were “cured,” while 37.6% completed their medication. In total, 90.2%, which equals the WHO 2011–2015 updated cure rate target of 87.0%, was achieved from 2012 to 2016 at the treatment site of the Metropolitan Hospital. This study's results suggest an improvement in treatment outcome. However, further studies are required to explain the factors associated with unfavourable outcomes, such as death and treatment failures at this TB treatment site as these could threaten the success of the National TB Control Programme in future.

Default rate throughout the entire period was 0.9% compared to the WHO interventional target of 5.0%. This has been achieved through establishment of zonal treatment sites to maximize reach and supervision of adherence to medication. For example, all TB clients who accessed diagnostic services at our treatment site were referred back to treatment sites within their respective locations. Only clients within the Cape Coast Metropolitan Assembly were offered treatment, and this could have led to such an outcome. In addition, the treatment site adopts a facility based DOTS backed by a community strategy involving intensive defaulter tracing through house address and telephone number calling. These could have contributed to the increased adherence. The study, however, documented an 8.5% death rate in total. Death rate ranges from 7% to 35% worldwide [15–17]. Our results and interactions with nursing staff suggest that clients could have died at home due to lack of proper care and poor adherence to treatment regimen. Counselling, sustained supervision, home visits, and health education have been used successfully as interventions to reduce defaulter rate/loss to follow-up of TB patients [18].

This study showed that sex was not significantly associated with treatment outcome but two times more of the males died compared to females. Males are known to have poor health seeking behavior than females and usually seek medical attention late at advanced stages of the disease. Males are known to have poor adherence to treatment and often default treatment compared to females [19, 20].

Sputum smear-positive clients are infectious to close contacts and the general population throughout the treatment period. Following commencement of treatment, patients have a reduction in AFB load; however, they continue to expel viable bacilli for a variable period of time. In the present study, 8.4% [(sc: 9, 1+: 6, 2+: 1, and 3+: 2)/213] still had AFB after 2-3 months of treatment. At the end of the 5th month up till the 8th month, 1.9% [(sc: 1, 1+: 2, and 3+: 1)/213] remained not cured and would continue to expel viable bacilli. Treatment failure was seen in the age groups of 20–29 (0.9%, 2/213) and 50–59 (0.5%, 1/213) years. Two clients were lost to follow-up, one each in the age groups of 20–29 and ≥ 60 years, respectively. Our findings are contrary to a study in Thailand, which showed that an age ≥ 60 years significantly correlated with treatment interruption and/or treatment failure [21]. Furthermore, the assertion in other studies [21–23] that an older age is a risk factor for death in pulmonary TB is incongruent with findings of this study. Out of 30 who died at home in this study, only one person was ≥60 years old. Further studies are required in TB clients on DOTS to bring finality to inconsequential outcomes.

This study found 13.6% of clients on DOTS strategy within the period to be infected with HIV. Out of this, 6.1% was among the age group of 40–49 years. Although males constituted the majority on DOTS, HIV prevalence of females, 6.6%, almost equaled that of males, 7.0%.

It is known that increased risk, as well as multidrug-resistant (MDR) tuberculosis, is mostly found in clients who are HIV positive. In this study, two MDRs were identified: one was HIV positive, while the other was HIV negative. Although studies in Ethiopia have shown high default likelihood among TB/HIV coinfected clients [19, 20], our study did not show any default among this category. In total, the number of clients with TB/HIV coinfection was 15 (7.0%). TB/HIV coinfection was highest (7/15) among the age group of 40–49 years. The potential of this age group being economically and sexually active cannot be substantiated without further studies. Since the overall number of HIV positive patients in the study is small, we could not establish the impact of HIV on sputum smear conversion. Literature search is yet to establish any effect in clients with TB/HIV coinfection [9, 24, 25]. Overall, the DOTS strategy has performed well, requiring continuous modifications and reviews to enhance its outcome.

## 5. Conclusion

We found a high treatment success rate among clients treated at our site. The relevant strategies to ensure early detection and treatment are under implementation. As expected, administration of medication on the wards is supervised by trained staff. Provision has also been made for trained staff to administer the DOTS and SLDs in communities where clients live. The information currently lacking is the potential negative sociodemographic/economic factors that result in unfavourable outcomes not controlled by the DOTS strategy.

## Figures and Tables

**Table 1:** Recommended DOTS treatment regimen for each category of tuberculosis in Ghana.

Patient category	Definition	Initial phase treatment^1*∗*^	Continuation phase treatment^*∗*^
		Daily (28 doses/month)	Daily (28 doses/month)
	*All new cases*		
I	(i) New smear-positive	*2 (HRZE)* ^2^ = 56 doses of HRZE	*4 (HR)* = 112 doses of HR
	(ii) New smear-negative PTB		
	(iii) Concomitant HIV disease		
	(iv) Extrapulmonary TB		

	*Previously treated sputum smear-positive PTB*		
II	(i) Relapse	*2 (HRZE)S + 1 (HRZE) *= 84 doses of HRZE + 56 doses of S	*5 (HRE)* = 140 doses of HRE
	(ii) Treatment after interruption		
	(iii) Treatment failure		

III^3^	*Children below 12 years*	*2 (HRZ)* = 56 doses of HRZ	*4 (HR)* = 112 doses of HR

*Table 1 is adopted from the National Tuberculosis Programme (NTP) Training Manual, 2012*. ^1^ *Direct observation of treatment intake is required and always in regimens including rifampicin*. ^*2*^ *Streptomycin may be used instead of ethambutol. In meningitis, ethambutol should be replaced by streptomycin*. ^3^ *In children with meningitis, add streptomycin in the initial phase*. Category I: new clients; category II: previously treated clients; category III: children < 12 years. *TB drugs and codes*: H: *isoniazid;* R: *rifampicin;* Z: *pyrazinamide;* S: *streptomycin;* E: *ethambutol*. *Codes for Fixed Drug Combinations (FDC)*: (HR): isoniazid + rifampicin; (HRZ): isoniazid + rifampicin + pyrazinamide; (HRZE): isoniazid + rifampicin + pyrazinamide + ethambutol.  ^*∗*^ *The administration of these drugs has been expansively explained in the 2012 edition of the NTP Training Manual.*

**Table 2:** Age and sex distribution of clients from 2012 to 2016.

Age group (years)	Sex of clients^*∗*^		Total^*∗∗*^
	Female (%)	Male (%)	
<10	2 (0.9)	—	2
10–19	5 (2.3)	6 (2.8)	11
20–29	12 (5.6)	23 (10.8)	35
30–39	14 (6.6)	30 (14.1)	44
40–49	9 (4.2)	42 (19.7)	51
50–59	9 (4.2)	31 (14.6)	40
≥60	3 (1.4)	27 (12.7)	30

*Total*	*54 (25.4)*	*159 (74.6)*	*213*

^**∗**^Significantly *more males than females were recruited for treatment *(*χ* ^2^ = 16.9, *p* = 0.013). ^*∗∗*^Total number of clients on the *Directly Observed Treatment Short-course* (DOTS).

**Table 3:** Pretreatment sputum smear grade and treatment outcome among clients.

	Pretreatment sputum smear grade (%)					Total
	Negative	Scanty (sc)	(1+)	(2+)	(3+)	
*Age range (years)*						
<10	2 (0.9)	—	—	—	—	2
10–19	4 (1.9)	1 (0.5)	2 (0.9)	1 (0.5)	3 (1.4)	11
20–29	9 (4.2)	2 (0.9)	4 (1.9)	8 (3.8)	12 (5.6)	35
30–39	17 (8.0)	4 (1.9)	3 (1.4)	10 (4.7)	10 (4.7)	44
40–49	16 (7.5)	4 (1.9)	13 (6.1)	4 (1.9)	14 (6.6)	51
50–59	17 (8.0)	5 (2.3)	6 (2.8)	4 (1.9)	8 (3.8)	40
≥60	22 (10.3)	—	—	2 (0.9)	6 (2.8)	30
* Total*	*87 (40.8)*	*16 (7.5)*	*28 (13.1)*	*29 (13.6)*	*53 (24.9)*	*213*
*Sex*						
Female	25 (11.7)	6 (2.8)	4 (1.9)	7 (3.3)	12 (5.6)	54
Male	62 (29.1)	10 (4.7)	24 (11.3)	22 (10.3)	41 (19.2)	159
* Total*	*87 (40.8)*	*16 (7.5)*	*28 (13.1)*	*29 (13.6)*	*53 (24.9)*	*213*
*Treatment outcome*						
Cured	—	13 (6.1)	25 (11.7)	27 (12.7)	45 (21.1)	110 (51.6)
Completed	80 (37.6)	—	—	—	—	80 (37.6)
Treatment failure	—	1 (0.5)	2 (0.9)	—	—	3 (1.4)
Lost to follow-up	1 (0.5)	—	—	1 (0.5)	—	2 (0.9)
Died at home	6 (2.8)	2 (0.9)	1 (0.5)	1 (0.5)	7 (3.3)	17 (8.0)
Died at Chest Ward	—	—	—	—	1 (0.5)	1 (0.5)
* Total*	*87 (40.8)*	*16 (7.5)*	*28 (13.1)*	*29 (13.6)*	*53 (24.9)*	*213*

Negative: *no Acid-Fast Bacilli (AFB) seen in at least 100 fields*; scanty (sc): *1–9 AFB found in 100 fields*; (1+): *10–99 AFB found in 100 fields*; (2+): *1–10 AFB found per field in at least 50 fields*; (3+): *more than 10 AFB per field in at least 20 fields*.

**Table 4:** HIV infection status among TB clients.

	HIV infection		Total
	Positive (%)	Negative	
*Sex*			
Male	15 (7.0)	144	159
Female	14 (6.6)	40	54
*Age*			
<10	—	2	2
10–19	1 (0.5)	10	11
20–29	4 (1.9)	31	35
30–39	6 (2.8)	38	44
40–49	13 (6.1)	38	51
50–59	2 (0.9)	38	40
≥60	3 (1.4)	27	30
*Year*			
2012	3 (1.4)	41	44
2013	10 (4.7)	45	55
2014	7 (3.3)	41	48
2015	6 (2.8)	38	44
2016	3 (1.4)	19	22
